# Frequency Noise Properties of Lasers for Interferometry in Nanometrology

**DOI:** 10.3390/s130202206

**Published:** 2013-02-07

**Authors:** Jan Hrabina, Josef Lazar, Miroslava Holá, Ondřej Číp

**Affiliations:** Institute of Scientific Instruments, v.v.i., Academy of Sciences of the Czech Republic, Královopolská 147, Brno 61264, Czech Republic; E-Mails: joe@isibrno.cz (J.L.); hola@isibrno.cz (M.H.); ocip@isibrno.cz (O.C.)

**Keywords:** nanometrology, laser noise, interferometry, nanopositioning, AFM

## Abstract

In this contribution we focus on laser frequency noise properties and their influence on the interferometric displacement measurements. A setup for measurement of laser frequency noise is proposed and tested together with simultaneous measurement of fluctuations in displacement in the Michelson interferometer. Several laser sources, including traditional He-Ne and solid-state lasers, and their noise properties are evaluated and compared. The contribution of the laser frequency noise to the displacement measurement is discussed in the context of other sources of uncertainty associated with the interferometric setup, such as, mechanics, resolution of analog-to-digital conversion, frequency bandwidth of the detection chain, and variations of the refractive index of air.

## 1. Introduction

Laser interferometry is the fundamental measuring technique for length and all dimensional quantities in fundamental metrology as well as in demanding industrial applications. The measurement range of laser interferometry covers the nano-world in the most precise devices like metrological scanning probe microscopes (SPMs), middle-sized objects, measured via coordinate measurement machines (CMMs), and up to kilometer distances in long-distance measurements [1–5]. The design of interferometric systems needs a great care when demands for precision are high. The whole arrangement of a laser interferometer consists of several key components—a laser source which powers the optical section of the interferometer, generating precise wavelength acting as a “ruler scale” in distance measurement, optics and mechanics designed according to the application, and finally the detection chain for acquisition, processing and evaluation of the interference signal including electronic hardware and software (demodulation and linearization techniques) [6–14]. Each component plays a role in the evaluation of the overall uncertainty.

### 1.1. Laser Source Noise Properties

As the laser interferometer relies on coherent laser light and actually counts discrete wavelengths, the intensity and mainly frequency noise of the laser source can significantly influence the uncertainty of the interferometric measurement. These properties have to be taken into consideration—especially frequency noise of the laser is directly transferred into the noise of the measured distance (quantity).

#### 1.1.1. Slow Frequency Noise—Drift

The most common type of lasers used in practical metrology of length are the relatively cheap He-Ne lasers operating at 633 nm wavelength. They offer relatively good long-term frequency stability up to range of 10^−11^ when frequency-stabilized by saturated absorption of molecular iodine [15–17]. Stabilization to the active line in Ne offers stability at the 10^−8^ level which is fully sufficient for measurements done under atmospheric conditions [18–21]. The main limitation in this case are the fluctuations of the refractive index of air (which can be controlled down to the range of approx. 10^−7^) [22–24]. The main disadvantage of He-Ne lasers is their limited power. Especially in multidimensional systems like CMM machines, where more interferometers are supplied from a single laser source, the He-Ne laser power can be a limiting factor. Over several years frequency doubled Nd:YAG lasers have become popular in metrology of length. They offer more power able to feed many measuring axes, a slightly shorter wavelength of 532 nm means better resolution and also better noise properties can be observed. The long-term frequency stability of the Nd:YAG lasers close to the 10^−14^ level for an integration time of 1,000 s can be achieved in optical setups where saturation spectroscopy in molecular iodine techniques are implemented [25–32]. In less demanding setups (interferometry under atmospheric conditions) for example, simple linear spectroscopy in molecular iodine can do the job to reduce long-term drift and offers frequency stability of 5 × 10^−9^ for 100 s [33–38].

#### 1.1.2. Frequency Noise

Faster frequency fluctuations of the laser source can degrade the interferometric measurement [39,40]. Especially in applications that cover nanometrology, where the demands are the highest and high-speed scanning and, thus large bandwidth are needed. This is why in this contribution we have focused especially on the measurement of frequency noise of various laser sources intended for use in laser interferometry. The laser noise analysis will be described in the experimental section together with description of the experimental setup. The problem of laser noise is related also to the frequency bandwidth, other properties of the detection system and length of the measuring arm of the interferometer.

### 1.2. Interferometer Configuration

Any interferometric arrangement with reference and measuring beam paths is sensitive to the wavelength of the laser source in relation to the difference in their optical lengths. Low-coherent or white-light interferometry exploits this effect. The center of a white-light fringe gives the information about balance between the two optical paths [41,42]. This is valid also for Michelson interferometer designed for measurement of displacement with a highly coherent laser source subject to optical frequency noise. Variations of the laser optical frequency seen either as a long-term drift (instability) or a frequency (phase) noise contribute to the measurement uncertainty more, when there is a larger difference between the measuring and reference optical lengths and do not contribute at all when the arms are precisely balanced [5,40,43].

A similar effect in nature is caused by fluctuations of the refractive index of air. This affects the speed of light in atmospheric conditions and thus the conversion of optical frequency into wavelength, which is actually what the interferometer counts. The value of refractive index can be evaluated from the parameters of atmosphere down to range of 10^−7^[22–24] or more or less effectively compensated [44–50].

### 1.3. Resolution of A/D Converters and Frequency Bandwidth

**Figure 1.**
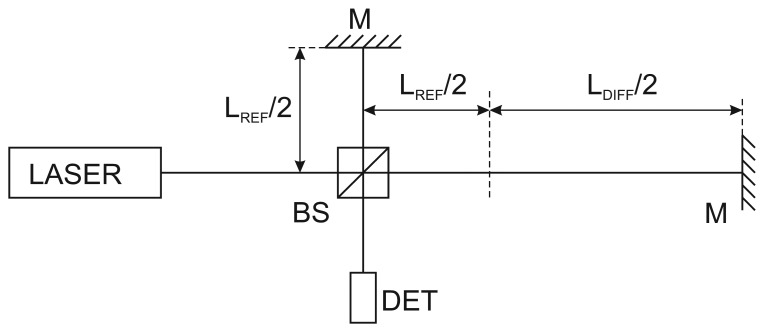
Principal schematic of a Michelson interferometer; BS—beamsplitter, M—mirrors, DET—photodetector, L_REF_—optical length of reference arm, L_DIFF_—difference distance between optical lenghts of reference and measuring arm of the interferometer.

From the point of view of the interference signal processing there comes the resolution of analog-to-digital converters in the detection chain. An interference fringe signal is sampled with this resolution and resolution of A/D converters is sometimes interpreted as the resolution of the interferometer. A dependency between A/D resolution and simple Michelson interferometer resolution is in Table 1 for 532 and 633 nm wavelength respectively. The resolution (minimal detectable position change of the measuring mirror) can be evaluated by Equation (1):

(1) $$L_{\text{MIN}}=\frac{\lambda }{2^{x}\times n}$$

where *λ* is wavelength of incident light, *x* is bit-resolution of the analog-to-digital converter and finally *n* is the number of measuring beams passing through the measured path in a multipass configuration (in case of setup like at Figure 1 n = 2). The resolution of the interferometer is influenced by the laser and other sources of noise and interference and has to be considered together with the desired frequency (speed) bandwidth of the system. Multipass setup may be an option.

This approach may lead to unrealistic expectations. For example for 18-bit resolution of A/D converter we could obtain 1 pm displacement resolution of the interferometer, but when there is a difference in lengths of reference and measuring arms, frequency fluctuation above this limit will also influence the result (not to speak about the refractive index), so the frequency/phase noise effects have to be considered. Next, especially in case of higher bit resolution of the A/D converter other sources of the noise start to influence the measurement and should be considered and precisely controlled. For example noise of the used electronics, noise floor of the photodetectors and proper electrical grounding and shielding should be taken into account. These properties are mainly of technical character and their detail analysis depends on concrete solution of the interferometric setup.

The frequency bandwidth of the whole detection chain plays also a role. This parameter is closely related to the spectral properties of the laser source and every source of variations (including fluctuations of refractive index of air, acoustic noise, *etc.*) has to be judged from the point of view of their speed and the required speed of the measurement.

From the point of view of the overall uncertainty of the measurement also interferometer nonlinearity errors should be addressed and taken into account. The nonlinearity of the phase detection can be effectively suppressed by proper linearization methods published before [8–11,13,14,43].

## 2. Analysis of the Interferometer

Considering the Michelson interferometer optical setup as in Figure 1, from the point of view of the frequency noise and frequency fluctuations the key parameter is the length of L_DIFF_—difference between optical lengths of reference and measuring arm of the interferometer. The influence of the frequency noise level will be higher in case of higher optical length difference between both arms. When this contribution reaches the level of *λ*/2 the interferometer loses the count of fringes—the interferometer goes beyond the coherence length of the laser. This also means that interferometers with arms of similar lengths (for example for measuring within a short range) will be less sensitive to the frequency fluctuations of the laser source.

**Figure 2.**
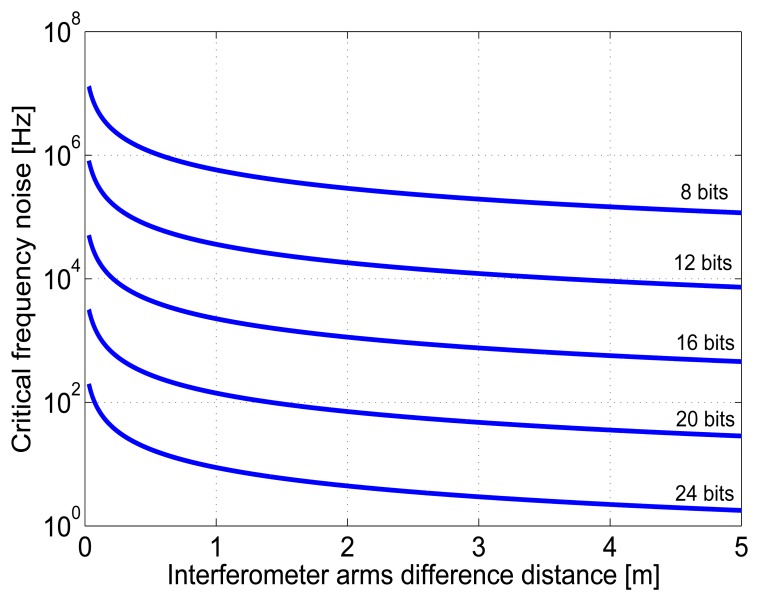
Length difference between the measuring and reference arms of the interferometer and corresponding limiting level of frequency fluctuations for 532 nm wavelength. (resolutions of the A/D conversion from top: 8, 12, 16, 20, 24 bits).

When we want to define a sensitivity of the interferometer to the frequency noise of the laser, we can use an expression of minimal frequency change which will influence the result of measurement (or which will exceed the quantization noise/resolution of the A/D converter). A length change Δ*L* seen at the output of the system caused by frequency shift of the laser light can be expressed as Equation (2):

(2) $$\Delta L=L_{\text{DIFF}}\times \left(\frac{f_{0}}{f_{\text{ACT}}}-1\right)$$

where *L_DIFF_* is distance difference between reference and measuring arms, *f_ACT_* is actual (shifted) optical frequency and *f*_0_ is the central optical frequency of the laser source (*f*_0_*= c/λ*). A dependency between the length difference between the interferometer arms and a limiting level of frequency noise exceeding the quantization noise is in Figure 2 for different resolutions of the A/D conversion and the wavelength 532 nm of the laser.

Next aspect related with the laser source frequency noise properties which should be considered is the frequency bandwidth of the system. A maximal speed of displacement of the interferometer measuring path *v_MAX_* which can be detected by the system from Figure 1 can be simply described as in Equation (3):

(3) $$v_{\text{MAX}}=\frac{\lambda \times f_{SA}}{4}$$

where *λ* is wavelength of incident light and *f_SA_* is sampling frequency of the analog-digital converter. The example values of these reacheable moving speeds for 532 and 633 nm wavelengths and three different sampling speeds are shown in Table 2.

In this contribution we concentrate on one of the sources of uncertainty in interferometry related to the laser noise. Considering facts described above we decided to investigate the short-term amplitude and also frequency noise properties of several different laser sources and tried to compare and evaluate their properties.

## 3. Experimental Section

We set up an experimental arrangement for simultaneous measuring of both amplitude and short-term frequency noise of several single-frequency He-Ne lasers and also frequency doubled Nd:YAG lasers. The optical power amplitude fluctuations were measured directly by a photodetector. Frequency noise of the laser radiation was measured with the help of a passive Fabry-Perot cavity used as an optical frequency discriminator. This cavity contained a mirror holder equipped with piezoelectric element which allowed tuning of the cavity length. The length of tunable Fabry-Perot cavity was in the next step stabilized by slow servo-loop (τ ∼3 s) to the value of investigated laser optical frequency so the frequency of the laser matched the middle point of the slope of the resonant transmission curve of the cavity. In this regime the cavity operated as a frequency discriminator suitable for fast laser noise fluctuations measurement. The free spectral range of the cavity was 2 GHz and measured linewidth of the cavity was 25 MHz. While the output signal of this frequency discriminator contained both the amplitude and frequency noises, the amplitude noise measured directly by photodetector and frequency fluctuations investigated through the cavity were recorded simultaneously. This gave us an opportunity to subtract the influence of amplitude noise from the frequency discriminator output. The cavity was inserted into an evacuated and thermal-shielded chamber to suppress the influences of refractive index of air fluctuations.

**Figure 3.**
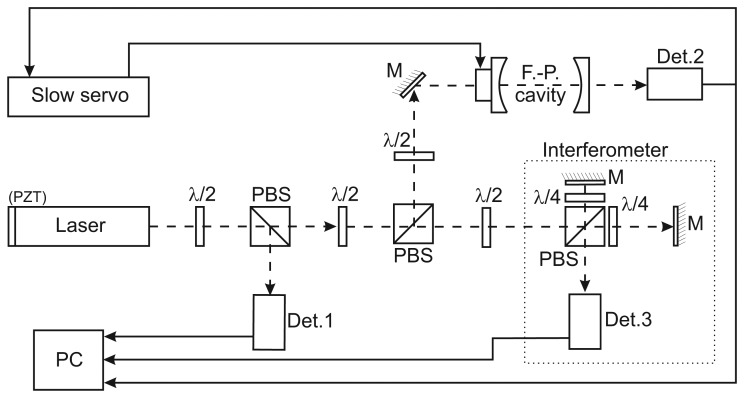
Schematic of the experimental setup for amplitude and frequency noise measurements. PBS-polarizing beam splitters, M-full reflective mirrors, Det-photodetectors (Det1 measuring amplitude noise, Det2 frequency noise throught the Fabry-Perot cavity, Det3 frequency noise throught the interferometer). PZT tuning option is included only in L1 laser and it was used only for evaluation of correct function of the frequency discriminators before the experiment itself.

The correct operation of the frequency discriminator was evaluated by the comparison of results from second frequency noise measurement through the reference Michelson interferometer with homodyne quadrature detection. The interferometer reacts to variations of the input laser frequency by variations of the phase of the signal on the output of the quadrature detection unit so it can be seen as another instrument of a frequency discriminator operating within the range of a single interference fringe. The key advantage of this approach is only negligible influence of amplitude fluctuations of the laser power to the output signal because the detection system response to these fluctuations is a few times smaller in comparison to frequency noise. We used a Michelson interferometer in four-pass configuration and a flat mirror reflector. The optical length difference between reference and measurement arm of the interferometer was 2 m (0.5 m mechanical distance) and the measurement were performed in thermal-shielded box to minimize the influence of the environmental conditions. Both measurements (with the help of the Fabry-Perot cavity and also with the help of interferometer) were performed in the same time to have a possibility to compare the results. One of the tested laser was equipped with a resonator mirror holder supplemented with piezoceramic element (PZT). This option enable us to frequency modulate this laser throught the voltage change of this PZT. This PZT was modulated by sinusoidal signals of different frequencies (1–30 kHz) and amplitudes to have a possibility to separate the modulation influence from the other frequency noise sources and results from cavity and also interferometer measurements were compared (frequency widths of this modulation were selected few orders higher than the noise background of the system itself). Results show very good correlation between 1st harmonics of the modulation signal. Measured signals were simultaneously recorded with the digital acquisition card with the 16 bit resolution and 600 kSa/s per channel sampling. The whole experimental setup schematic is in Figure 3).

The amplitude and frequency noise was measured for different free-running (unstabilized) laser sources (list of the tested lasers is at Table 3). However L5 commercial general purposes laser head contains thermal stabilization by the two-mode stabilization technique [18,19,51].

L1 (L2) laser is an ultra-stable ring Nd:YAG laser intended for metrology applications. It offers a “noise-eater” option which means switchable internal filter of amplitude noise. Measurement was done with the filter activated and also deactivated (highlighted as L1 and L2 respectively). L3 laser is an ultra-stable diode-pumped ring laser with an intracavity frequency doubling. Both L1 and L3 are primarily intended for saturated sub-Doppler spectroscopy in iodine vapor at 532 nm wavelength and are designed to operate as laser optical frequency standards [21,25–32]. L4 laser is a simple diode pumped solid state laser (DPSSL) with alignment-free monolithic resonator equipped with slow thermal frequency tuning option which allows linear absorption spectroscopy frequency stabilization technique. Some results of the long-term frequency stability of this laser stabilized by linear absorption technique are summarized in [5,36,38]. L5 and L6 lasers are commonly used commercial single-mode He-Ne lasers from different producers, L5 is equipped with thermal two-mode frequency stabilization, L6 is completely free-running. L7 is an approximately 25 years old commercial He-Ne laser, where degradation of the laser tube during long time period can be expected. Finally, L8 is He-Ne-I_2_ laser—iodine stabilized optical frequency standard for 633 nm [16,17].

## 4. Results and Discussion

**Figure 4.**
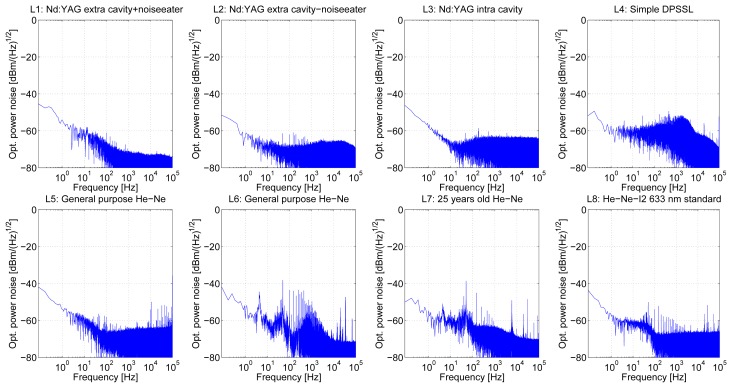
Optical power (amplitude) noise measurements of tested laser heads.

Amplitude noise measurements (in range 0–100 kHz) were performed for free-running lasers directly by measurement of optical power fluctuations using the low noise photodetector after few hours of stable operation of all of the laser heads (Figure 4). The detection chain was not completely free of unwanted interfering electric signal with 20 kHz frequency coming from the switched laser power supplies. Another 50 Hz frequency component from the power supply network could not be completely avoided so these frequency components are present in measured spectra.

The best results were obtained with L1(2) and L3 (both of them designed for optical frequency standards) with noise floor level below −70 dBm/Hz^1/2^ and −62 dBm/Hz^1/2^, respectively, within the investigated bandwidth. L4 (simple DPSSL) shows low frequency fluctuations in the region of 0–10 kHz, caused most likely by acoustic noise interference due to mechanical vibrations of fan-cooled laser head. In case of He-Ne lasers the best result was obtained with L8 (He-Ne-I_2_ standard), with noise floor at −65 dBm/Hz^1/2^ and few harmonics (about −55 dBm/Hz^1/2^), especially in the region of 35 kHz. Another of our He-Ne lasers shows amplitude fluctuations in range of −45 to −50 dBm/Hz^1/2^. We assume that these fluctuations were mainly caused by electric interference from the switched power-supplies and in case of L7 also by ageing of old laser tube.

**Figure 5.**
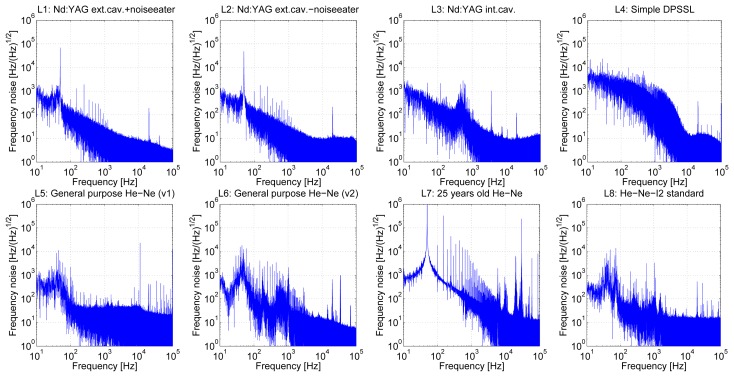
Frequency noise measurements of tested laser heads (Fabry-Perot cavity).

The frequency noise measurements (Figure 5) were done through the Fabry-Perot cavity frequency discriminator at the same sampling speeds as amplitude noise measurements (600 kSa/s) after the evaluation of the proper operation of the discriminator.

The RMS (root mean square) values of the frequency noise were calculated within different frequency bandwidths. Table 4 shows RMS values of frequency noise for spectral components above 10 Hz (to suppress an influence of control servo-loop of the Fabry-Perot cavity), Table 5 shows noise levels above 100 Hz to suppress the influence of 50 Hz spectral component present due to electric interference.

Considering the values above and the desired frequency bandwidth of the system, it is possible to evaluate the resulting noise of the measured displacement induced by the laser noise by the modified Equation (2). When Δ*f* is the RMS value of the frequency noise within the considered bandwidth, the corresponding RMS value of the length variations at the output of the interferometer (“length noise”) will be:

(4) $$\Delta L=L_{\text{DIFF}}\times \left(\frac{f_{0}}{f_{0}-\Delta f}-1\right)$$

where *L_DIFF_* is length difference between reference and measuring paths of the interferometer and *f_0_* is the central optical frequency of the laser source. Some selected noise values for different paths differences and for different levels of frequency noise were computed and are shown in the Table 6.

When we put together calculated values from Table 6 and measured results from Tables 4 and 5 we can estimate a contribution of the laser frequency noise to the overall uncertainty of the interferometric distance measurement. For considered frequency bandwidth from 100 Hz–100 kHz and interferometer paths length difference 0.01 m we get RMS noise of the measured length of 8.30e-14 m for the L1 laser (4.68 kHz/(Hz)^1/2^) to 5.25e-12 m length noise for the L7 laser (296 kHz/(Hz)^1/2^). For the considered system frequency bandwidth from 100 Hz–300 kHz and interferometer paths length difference 1 m we get RMS noise of the measured length of 8.83e-12 m for the L1 laser (4.98 kHz/(Hz)^1/2^) to 5.99e-10 m length noise for the L7 laser (337.81 kHz/(Hz)^1/2^). Consequently, great care should be taken to frequency noise contribution to the measurement uncertainty.

## 5. Conclusions

Our contribution deals with the interferometric measurements of lengths and discussion about aspects which influence the measurement results. We mainly concentrated on the short-term frequency noise of the laser head, which is one of the key parameters of the interferometer. In the experimental section the amplitude and frequency noise of different laser sources intended for interferometry were measured. The experimental setup with frequency discriminator represented by Fabry-Perot cavity with length stabilized through the slow servo-loop to the frequency of the investigated laser optical frequency was assembled. Measurements of amplitude and frequency noises were done simultaneously with using the high-speed digitization card. The correct operation of frequency discriminator was tested with the help of additional modulation of the laser frequency and the second part of the optical setup—the Michelson interferometer. The frequency measurements results show very good correlation between Fabry-Perot measurements and interferometer measurements. The best results of the amplitude noise perform at 532 nm Nd:YAG laser standards (L1,2 and L3), in case of He-Ne and 633 nm wavelength the best laser is L8 (He-Ne-I_2_ standard). All of these lasers are intended to operate as laser standards for realization of fundamental etalons of length at 532 and 633 nm respectively in the laboratory environment. Their design is more precise, these lasers are more expensive in comparison to other tested laser sources. L4 (a simple DPSSL) contains internal servo loops for driving the laser and also a fan for cooling of the laser which caused huge frequency variations in the low frequency range. The worst frequency stability results corresponded to the L7 laser (an old one), mainly due to degradation due to the long history of this laser, old construction of the laser itself and its power supply. We computed achievable RMS values of the “length noise” for different levels of the frequency noise of the laser together with several measuring differences and reference paths of the interferometer. These results give us important information about the influence of the laser head's frequency noise properties to the interferometric measurement and the resolution and uncertainty contribution to the system. Exceptional interest should be taken on them especially for the setups with large interferometer paths difference. The next aspects we investigated are the resolution limit and bandwidth of the interferometer and its detection chain. We expect that greatest contributors to the frequency noise in the recordings are the switching power supplies of the laser heads. The results show better performance in frequency stability for measuring systems with Nd:YAG lasers in comparison to traditionally used He-Ne ones, especially for higher frequencies. This corresponds to measurements of long-term frequency stabilities which were done before [5,26–28]. Higher power available with Nd:YAGs lasers is not only high enough for feeding all of the interferometers needed especially in case of multidimensional systems but high power level incident on the photodetectors needs lower gain of the following amplifiers and thus improves the noise performance of the detection chain.

## Figures and Tables

**Table 1.** Dependency between the resolution of A/D conversion and resolution (minimal detectable distance) of the interferometer for the 532 and 633 nm wavelengths respectively.

	**Resolution of the interferometer [nm]**	
A/D resolution	*λ* = 532 nm	*λ* = 633 nm
12 bits	6.49	7.73
14 bits	1.62	1.93
16 bits	0.41	0.48
18 bits	0.10	0.12
20 bits	0.025	0.030
22 bits	0.0063	0.0076
24 bits	0.0016	0.0019

**Table 2.** Maximal detectable speeds of distance changes *vs. f_SA_* of ADC.

***f****_SA_***[kHz]**	***v****_max_***[m/s],*λ*= 532 nm**	***v****_max_***[m/s],*λ*= 633 nm**
0.1	2.66e-5	3.16e-5
1	2.66e-4	3.16e-4
10	2.66e-3	3.16e-3
100	2.66e-2	3.16e-2
300	7.98e-2	9.48e-2

**Table 3.** A list of tested laser sources.

**Laser no.**	**Description**	**Optical power [mW]**
L1	Nd:YAG external cavity doubling, +noiseeater	20
L2	Nd:YAG external cavity doubling, –noiseeater	20
L3	Nd:YAG internal cavity doubling	30
L4	Nd:YAG, Simple DPSSL (diode pumped solid state laser)	50
L5	He-Ne, general purposes, thermally stabilized	5
L6	He-Ne, general purposes	0.8
L7	He-Ne, 25 years old general purposes	0.2
L8	He-Ne-I_2_ laser standard	0.2

**Table 4.** RMS values of frequency noise *vs.* frequency bandwidth [kHz/(Hz)^1/2^]. Starting frequency of the considered band is 10 Hz.

	**Frequency noise bandwidth (from 10 Hz to …)**				

	**100 Hz**	**1 kHz**	**10 kHz**	**100 kHz**	**300 kHz**
L1	52.90	53.00	53.01	53.03	53.04
L2	50.70	50.83	50.85	50.91	50.94
L3	12.36	17.44	17.89	18.13	21.35
L4	73.42	95.56	96.80	96.86	96.87
L5	17.20	17.32	17.94	44.12	45.33
L6	33.88	35.55	36.53	39.32	39.39
L7	1,197.35	1,220.85	1,220.93	1,233.39	1,244.09
L8	24.37	24.71	25.13	25.35	26.00

**Table 5.** RMS values of frequency noise *vs.* frequency bandwidth [kHz/(Hz)^1/2^]. Starting frequency of the considered band is 100 Hz.

	**Frequency noise bandwidth (from 100 Hz to …)**			

	**1 kHz**	**10 kHz**	**100 kHz**	**300 kHz**
L1	3.14	3.37	3.60	3.72
L2	3.67	3.90	4.68	4.98
L3	12.31	12.92	13.26	17.42
L4	61.17	63.09	63.18	63.19
L5	2.8	5.11	40.63	41.94
L6	10.75	13.64	19.94	20.9
L7	238.38	238.79	296.00	337.81
L8	4.10	5.79	6.99	9.059

**Table 6.** Exemplary computed RMS length noise ([m]) dependency on interferometer paths differences and the frequency noise of the laser.

**L_DIFF_[m]**	**Δf [kHz]**			

	**1e1**	**1e2**	**1e3**	**1e4**
0.01	1.77e-13	1.77e-12	1.77e-11	1.77e-10
0.1	1.77e-12	1.77e-12	1.77e-10	1.77e-9
1	1.77e-11	1.77e-12	1.77e-9	1.77e-8

## References

[1] Korpelainen V., Seppa J., Lassila A. (2010). Design and characterization of MIKES metrological atomic force microscope. Precis. Eng..

[2] Jäger G., Gruenwald R., Manske E., Hausotte T., Fuessl R. (2004). A nanopositioning and nanomeasuring machine: Operation-measured results. Nanotechnol. Precis. Eng..

[3] Lazar J., Hrabina J., Sery M., Klapetek P., Cip O. (2012). Multiaxis interferometric displacement measurement for local probe microscopy. Cent. Eur. J. Phys..

[4] Kim J.A., Kim J.W., Park B.C., Eom T.B. (2006). Measurement of microscope calibration standards in nanometrology using a metrological atomic force microscope. Meas. Sci. Technol..

[5] Hrabina J., Lazar J., Klapetek P., Cip O. (2011). Multidimensional interferometric tool for the local probe microscopy nanometrology. Meas. Sci. Technol..

[6] Wu C.M., Su C.S., Peng G.S. (1996). Correction of nonlinearity in one-frequency optical interferometry. Meas. Sci. Technol..

[7] Yacoot A., Downs M.J. (2000). The use of x-ray interferometry to investigate the linearity of the NPL Differential Plane Mirror Optical Interferometer. Meas. Sci. Technol..

[8] Cip O., Petru F. (2000). A scale-linearization method for precise laser interferometry. Meas. Sci. Technol..

[9] Petru F., Cip O. (1999). Problems regarding linearity of data of a laser interferometer with a single-frequency laser. Precis. Eng..

[10] Eom T., Kim J., Jeong K. (2001). The dynamic compensation of nonlinearity in a homodyne laser interferometer. Meas. Sci. Technol..

[11] Rerucha S., Buchta Z., Sarbort M., Lazar J., Cip O. (2012). Detection of interference phase by digital computation of quadrature signals in homodyne laser interferometry. Sensors.

[12] Ottonelli S., Dabbicco M., de Lucia F., di Vietro M., Scamarcio G. (2009). Laser-self-mixing interferometry for mechatronics applications. Sensors.

[13] Olyaee S., Nejad S.M. (2007). Nonlinearity and frequency-path modelling of three-longitudinal-mode nanometric displacement measurement system. IET Optoelectron.

[14] Badami V.G., Patterson S.R. (2000). A frequency domain method for the measurement of nonlinearity in heterodyne interferometry. Precis. Eng..

[15] Quinn T.J. (1994). Mise-En-Pratique of the definition of the meter (1992). Metrologia.

[16] Navratil V., Fodrekova A., Gata R., Blabla J., Balling P., Ziegler M., Zeleny V., Petru F., Lazar J., Vesela Z. (1998). International comparisons of He-Ne lasers stabilized with I-127(2) at lambda approximate to 633nm (July 1993 to September 1995)—Part III: Second comparison of Eastern European lasers at lambda approximate to 633 nm. Metrologia.

[17] Hrabina J., Petru F., Jedlicka P., Cip O., Lazar J. (2007). Purity of iodine cells and optical frequency shift of iodine-stabilized He-Ne lasers. Optoelectron. Adv. Mat..

[18] Ciddor P.E., Duffy R.M. (1983). 2-Mode frequency stabilized He-Ne (633 Nm) lasers—studies of short-term and long-term Stability. J. Phys. E Sci. Instrum..

[19] Ciddor P.E., Bruce C.F. (1981). Long-Term stability of a thermally-stabilized He-Ne-Laser. Metrologia.

[20] Simmons J.D., Hougen J.T. (1977). Atlas of I2 spectrum from 19 000 to 18 000 Cm-1. J. Res. Natl. Bur. Stand. A Phys. Chem..

[21] Hrabina J., Jedlicka P., Lazar J. (2008). Methods for measurement and verification of purity of iodine cells for laser frequency stabilization. Meas. Sci. Rev..

[22] Edlen B. (1966). The refractive index of air. Metrologia.

[23] Zhang J., Lu Z.H., Menegozzi B., Wang L.J. (2006). Application of frequency combs in the measurement of the refractive index of air. Rev. Sci. Instrum..

[24] Lazar J., Hola M., Cip O., Cizek M., Hrabina J., Buchta Z. (2012). Displacement interferometry with stabilization of wavelength in air. Opt. Express.

[25] Galzerano G., Bava E., Bisi M., Bertinetto F., Svelto C. (1999). Frequency stabilization of frequency-doubled Nd:YAG lasers at 532 nm by frequency modulation spectroscopy technique. IEEE Trans. Instrum. Meas..

[26] Nevsky A.Y., Holzwarth R., Reichert J., Udem T., Hansch T.W., von Zanthier J., Walther H., Schnatz H., Riehle F., Pokasov P.V. (2001). Frequency comparison and absolute frequency measurement of I-2-stabilized lasers at 532 nm. Opt. Commun..

[27] Rovera G.D., Ducos F., Zondy J.J., Acef O., Wallerand J.P., Knight J.C., Russell P.S. (2002). Absolute frequency measurement of an I-2 stabilized Nd:YAG optical frequency standard. Meas. Sci. Technol..

[28] Lazar J., Hrabina J., Jedlicka P., Cip O. (2009). Absolute frequency shifts of iodine cells for laser stabilization. Metrologia.

[29] Picard S., Robertsson L., Ma L.S., Nyholm K., Merimaa M., Ahola T.E., Balling P., Kren P., Wallerand J.P. (2003). Comparison of I-127(2)-stabilized frequency-doubled Nd:YAG lasers at the Bureau International des Poids et Mesures. Appl. Opt..

[30] Galzerano G., Bava E., Bisi M., Bertinetto F., Svelto C. Frequency stabilization of frequency-doubled Nd:YAG lasers at 532 nm.

[31] Nyholm K., Merimaa M., Ahola T., Lassila A. (2003). Frequency stabilization of a diode-pumped Nd:Yag laser at 532 nm to iodine by using third-harmonic technique. IEEE Trans. Instrum. Meas..

[32] Holzwarth R., Nevsky A.Y., Zimmermann M., Udem T., Hansch T.W., von Zanthier J., Walther H., Knight J.C., Wadsworth W.J., Russell P.S.J. (2001). Absolute frequency measurement of iodine lines with a femtosecond optical synthesizer. Appl. Phys. B..

[33] Dai G.L., Pohlenz F., Danzebrink H.U., Xu M., Hasche K., Wilkening G. (2004). Metrological large range scanning probe microscope. Rev. Sci. Instrum..

[34] Cao H.J., Zang E.J., Zhao K., Zhang X.B., Wu Y.X., Shen N.C. (1998). Frequency stabilization of a Nd:YAG laser to Doppler-broadened lines of iodine near 532 nm. Proc. SPIE.

[35] Lazar J., Cip O., Cizek M., Hrabina J., Sery M., Klapetek P. Interferometer Controlled Positioning for Nanometrology.

[36] Hrabina J., Lazar J., Klapetek P., Cip O. (2011). AFM nanometrology interferometric system with the compensation of angle errors. Proc. SPIE.

[37] Otsuka J., Ichikawa S., Masuda T., Suzuki K. (2005). Development of a small ultraprecision positioning device with 5 nm resolution. Meas. Sci. Technol..

[38] Hrabina J., Lazar J., Cip O., Cizek M. (2010). Laser source for interferometry in nanotechnology. Soc. Photo Opt. Inst..

[39] Clivati C., Mura A., Calonico D., Levi F., Costanzo G.A., Calosso C.E., Godone A. (2011). Planar-Waveguide External Cavity Laser Stabilization for an Optical Link With 10(-19) Frequency Stability. IEEE Trans. Ultrason. Ferroelectr. Freq. Control.

[40] Lance A.L., Seal W.D., Labaar F. (1982). Phase noise measurement systems. ISA Trans..

[41] Buchta Z., Mikel B., Lazar J., Cip O. (2011). White-light fringe detection based on a novel light source and colour CCD camera. Meas. Sci. Technol..

[42] Buchta Z., Rerucha S., Mikel B., Cizek M., Lazar J., Cip O. (2012). Novel Principle of Contactless Gauge Block Calibration. Sensors.

[43] Yokoyama T., Araki T., Yokoyama S., Suzuki N. (2001). A subnanometre heterodyne interferometric system with improved phase sensitivity using a three-longitudinal-mode He-Ne laser. Meas. Sci. Technol..

[44] Lazar J., Cip O., Cizek M., Hrabina J., Buchta Z. (2011). Standing wave interferometer with stabilization of wavelength on air. TM Tech. Mess..

[45] Lazar J., Cip O., Cizek M., Hrabina J., Buchta Z. (2011). Suppression of air refractive index variations in high-resolution interferometry. Sensors.

[46] Birch K.P., Downs M.J. (1994). Correction to the updated edlen equation for the refractive-index of air. Metrologia.

[47] Lazar J., Hola M., Cip O., Cizek M., Hrabina J., Buchta Z. (2012). Refractive index compensation in over-determined interferometric system. Sensors.

[48] Quoc T.B., Ishige M., Ohkubo Y., Aketagawa M. (2009). Measurement of air-refractive-index fluctuation from laser frequency shift with uncertainty of order 10(-9). Meas. Sci. Technol..

[49] Smid R., Cip O., Lazar J. (2008). Precise length etalon controlled by stabilized frequency comb. Meas. Sci. Rev..

[50] Lazar J., Cip O., Cizek M., Hrabina J., Buchta Z. (2010). Interferometry with direct compensation of fluctuations of refractive index of air. Proc. SPIE.

[51] Balhorn R., Lebowsky F., Kunzmann H. (1972). Frequency stabilization of internal-mirror helium-neon lasers. Appl. Opt..

